# The effect of vitamin D supplementation on the glycemic control of pre-diabetic Qatari patients in a randomized control trial

**DOI:** 10.1186/s40795-019-0311-x

**Published:** 2019-10-10

**Authors:** Mohammed Al Thani, Eman Sadoun, Angeliki Sofroniou, Amin Jayyousi, Khaled Ahmed Mohamed Baagar, Abdulla Al Hammaq, Benjamin Vinodson, Hammad Akram, Zaid Shakoor Bhatti, Heba Samir Nasser, Vasiliki Leventakou

**Affiliations:** 1 Public Health Department, Ministry of Public Health, Doha, Qatar; 2 Biomedical Research Department, Ministry of Public Health, P.O. Box 42, Al Khaleej Str, Al Rumaila, Doha, Qatar; 3 Diabetes and Endocrine Department, Hamad Medical Corporation, Doha, Qatar; 4 Qatar Diabetes Association, Qatar Foundation, Doha, Qatar

**Keywords:** Vitamin D, Pre-diabetes, β-Cell function, Glucose metabolism, Middle East

## Abstract

**Background:**

Vitamin D deficiency is associated with indicators of pre-diabetes including, insulin resistance, β-cell dysfunction and elevated plasma glucose with controversial findings from current trials. This study aims to investigate the long-term effect of vitamin D on glucose metabolism and insulin sensitivity in pre-diabetic and highly vitamin-deficient subjects.

**Methods:**

One hundred thirty-two participants were randomized to 30,000 IU vitamin D weekly for 6 months. Participants underwent oral glucose tolerance test (OGTT) at 3-month intervals to determine the change in plasma glucose concentration at 2 h after 75 g OGTT (2hPCG). Secondary measurements included glycated hemoglobin, fasting plasma glucose and insulin, post-prandial insulin, indices of insulin sensitivity (HOMA-IR, Matsuda Index), β-cell function (HOMA-β, glucose and insulin area under the curve (AUC), disposition and insulinogenic indices), and lipid profile.

**Results:**

A total of 57 (vitamin D) and 75 (placebo) subjects completed the study. Mean baseline serum 25(OH) D levels were 17.0 ng/ml and 14.9 ng/ml for placebo and vitamin D group, respectively. No significant differences were observed for 2hPC glucose or insulin sensitivity indices between groups. HOMA-β significantly decreased in the vitamin D group, while area under curve for glucose and insulin showed a significant reduction in β-cell function in both groups. Additionally, HOMA-β was found to be significantly different between control and treatment group and significance persisted after adjusting for confounding factors.

**Conclusion:**

Vitamin D supplementation in a pre-diabetic and severely vitamin-deficient population had no effect on glucose tolerance or insulin sensitivity. The observed reduction in β-cell function in both placebo and vitamin D groups could be attributed to factors other than supplementation.

**Trial registration:**

NCT02098980, 28/03/2014 (www.clinicaltrials.gov).

## Background

Low levels of vitamin D has been identified as a major health issue globally. Current literature provides evidence for the association of hypovitaminosis D with various health conditions including bone health [1], cardiovascular disease [2], hypertension [3], cancer risk [4] and diabetes mellitus [5]. In particular, higher prevalence of low serum vitamin D [25-(OH)D] levels has been observed among type 2 diabetic patients [6] while higher levels of vitamin D [25-(OH)D] has been associated with lower risk of type 2 diabetes (T2DM) [7, 8]. The increasing rates of diabetes worldwide with diabetic patients reaching 400 million people, a number predicted to exceed 600 million by 2040, can justify the rapid shift of research interest towards prevention and treatment strategies for diabetes, including intervention with vitamin D [9].

Several possible mechanisms have been proposed to mediate the protective role of vitamin D against diabetes risk including alterations in the pancreatic β-cell function [10], insulin sensitivity [11] and systemic inflammation [12]. Despite the promising results from observational studies, intervention studies are more appropriate to provide insight on causality and develop hypothesis. Up to date, a number of randomized control trials (RCTs) have been conducted to evaluate the role of vitamin D supplementation in the pathogenesis of type 2 diabetes but results remain inconclusive. Recent systematic reviews and meta-analyses of clinical trials have found no association between vitamin D and glycemic indices and insulin resistance in patients with T2DM apart from a modest reduction on fasting glucose in some cases [13–15]. On the contrary, other have reported a small but positive effect of vitamin D supplementation on glycemic control, insulin resistance and glucose tolerance [5, 16, 17]. Regarding the role of vitamin D in the progression of T2DM definite conclusions cannot also be drawn. There are only few vitamin D intervention studies including pre-diabetic participants with inconsistent observations [18–20]. The disparity in the reported results could be attributed to various types (pills, drops, and vitamin D fortified foods) and doses of vitamin D supplements, participants’ vitamin D status (deficient/insufficient), other comorbid conditions, small study samples and possibly restricted time frame of supplementation.

Given the novel research focus on this topic and the conflicting evidence, we sought to expand current literature by including participants highly affected by the risk of developing diabetes [9] and at the same time deficient in vitamin D. These characteristics are likely to alter the efficacy of vitamin D supplementation, since better effects of supplementation have been observed in deficient patients rather than insufficient or sufficient [21]. This is the first placebo-control trial in the Middle East in a population with that aims to investigate the effect of 6 months vitamin D supplementation in pre-diabetic, vitamin-deficient subjects on glucose tolerance, insulin sensitivity and β-cell function in a relatively large sample.

## Methods

### Participants and study design

This intervention study was a 6-month, double-blind, randomized, placebo-controlled trial conducted at Hamad Medical Corporation (HMC) in Doha, Qatar. The research ethics board of HMC approved the protocol and the trial was registered at www.clinicaltrials.gov (no. NCT02098980). An informed consent was obtained from all participants at enrollment.

**Fig. 1:**
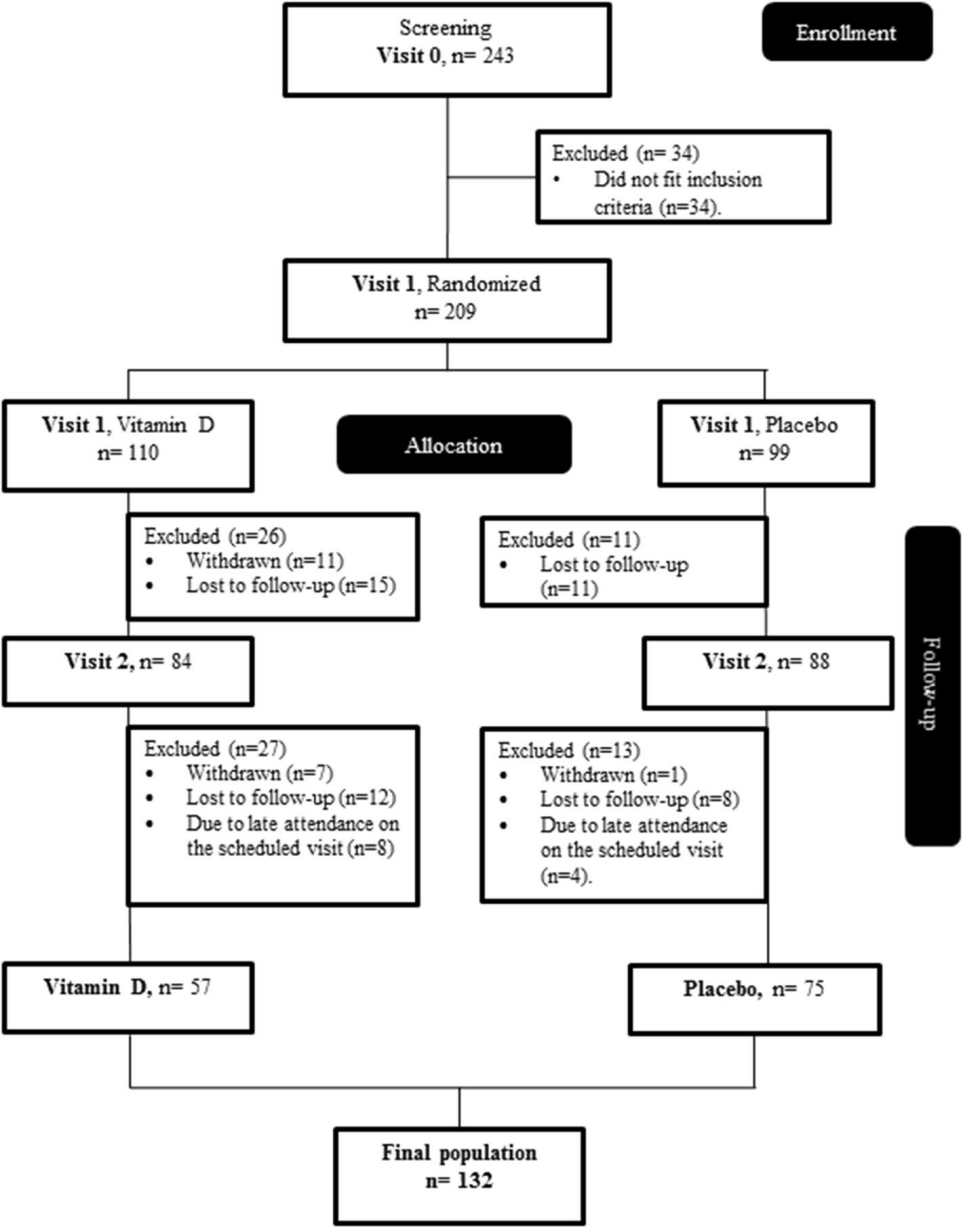
Flow diagram of the study participants

Men and women aged 18-75 of multicultural backgrounds were recruited via telephone calls and campaigns held at Qatar landmarks. A two-step process was used to screen for eligibility as shown in Fig. 1. In the first step, screening 1, eligibility was based on a finger prick HbA1c result (5.6-6.4%) indicating pre-diabetes [22]. During the second screening (visit 0), eligibility was based on physical and biochemical measurements, which included medical history, prescribed medication, height, weight, waist circumference, BMI, pulse, fasting glucose, HbA1c, cholesterol, triglycerides, liver function, blood analyses, insulin, C-peptide, serum 25(OH) vitamin D_3_, parathyroid hormone (PTH) and calcium.

Inclusion criteria were as follows: non-pregnant or lactating women, BMI < 40 kg/m^2^, serum 25(OH) vitamin D3 concentration < 30 ng/ml, fasting serum glucose < 7.0 mmol/L, HbA1c 5.6-6.4%. Subject inclusion also relied on the presence of one or more of the following: waist circumference of > 80 cm for females and > 90 cm for males [23], older than 40 years old, family history of diabetes in first-degree relative, previous history of gestational diabetes, history of high blood glucose or triglycerides and/or low HDL cholesterol. Participants were excluded if: fasting serum glucose ≥7.0 mmol/L, had history of renal failure or liver disease, serum urea or creatinine > 1.8 times the upper limit of normal (ULN), serum aspartate or alanine transaminase (AST, ALT) > 1.5 times ULN, use of medicine to treat diabetes or which influenced glucose metabolism at the time of screening, experienced a medical or surgical event requiring hospitalization within 3 months of randomization, and if they suffered from any condition affecting nutrient absorption (e.g. irritable bowel syndrome).

At visit 1, study participants were randomly assigned either the placebo or the vitamin D treatment at a dose of 30,000 IU/week (equivalent to 4000 IU per day) and were given supplements to cover a 3-month period. In this visit they completed the Finnish Diabetes Risk Score (FINDRISC) questionnaire [24], a 24-h recall food questionnaire, signed the consent form, and underwent their baseline 75 g OGTT (after overnight fast). All active participants visited the recruitment center two more times in 3-month intervals to complete 6 months of treatment and underwent the same physical and biochemical measurements as in visit 0 and 1.

### Randomization

ID numbers were assigned to subjects sequentially in the order in which their eligibility was ascertained and informed consent obtained. Subjects were randomly assigned to one of the 2 treatments in blocks of varying sizes. To achieve this, a set of sealed, opaque envelopes labelled with the ID number and containing the pre-assigned treatment code were created and assigned to subjects in the order they attended for visit 1. Subjects were randomized to control or vitamin D group opening the next available envelope. Randomization (done using the “@RAND” function on a Lotus 123 spreadsheet) and creation of the sealed envelopes was done by the research team at the recruiting center and a list of ID numbers and coded treatment assignments were kept in a secure web-based database.

### Biochemical measurements

The primary outcome was to determine the change in 2hPCG from baseline to end-point following supplementation with vitamin D. Participants underwent an oral glucose tolerance test (OGTT) at two 3-month intervals to determine the change in plasma glucose concentration at 2 h after 75 g OGTT (2hPCG). At each OGTT, plasma glucose was measured at four different time points, 30, 60, 90 and 120 min after glucose load. Secondary outcomes included lipid profile, glycated hemoglobin, fasting plasma glucose and insulin, post-prandial insulin, and insulin sensitivity derived from the Homeostatic Model of Assessment - Insulin Resistance (HOMA-IR) and Matsuda Index. B-cell function was measured by HOMA-β, disposition index (DI), insulinogenic index, and glucose and insulin area under the curve (AUC) from 0 to 120 min during OGTT at each time point (Baseline, 3 months, 6 months).

Serum glucose was measured using the hexokinase/G-6-PDH method (Abbott architect C systems analyzer). Chemiluminescence immunoassay (CLIA) technology (LIAISON Analyzer family) was used for the in vitro quantitative determination of insulin in human serum. Serum 25(OH) D were measured by the method of one-step immunoassay using Chemiluminescent Micro Particle Immunoassay (CMIA) technology (Abbott Architect iSystem analyzer).

### Statistical analysis

Data are presented as frequency (n, %) and means (± standard deviation, SD) and median (interquartile range, IQR) where appropriate. Paired t-test was used to test the within groups differences. For the non-parametric variables, we used the Wilcoxon signed rank test. Unpaired t-test was used to compare between two means of parametric continuous variables. Mann-Whitney U test was used to compare between two means of all non-parametric continuous variables. Primary outcomes were analyzed with ANCOVA using a general linear model with the change from baseline to endpoint for each outcome in both placebo and vitamin D groups adjusting for age, gender, baseline BMI and ethnicity. Bivariate associations were tested with Spearman rank correlation test to examine the strength of association between the differences (endpoint and baseline) of vitamin D. Multivariable linear regression models were implemented to test the association between primary and secondary outcomes and vitamin D difference between baseline and endpoint, after adjusting for the aforementioned confounding factors. Estimated associations were described with β-coefficients and 95% CI and *R*^2^ A *p*-value < 0.05 was considered statistically significant. All statistical analyses were performed using SPSS Statistics 19 software (SPSS Inc., Chicago, IL, USA).

Assessment of insulin sensitivity and β-cell function were estimated by the Homeostasis Model of Assessment-Insulin Resistance Index (HOMA-IR), the Homeostatic Model of Assessment- Beta (HOMA- β) and the Matsuda index and were calculated as follow [25, 26]:

$$ \mathrm{HOMA}-\mathrm{IR}=\frac{\left(\mathrm{Fasting}\ \mathrm{glucose}\times \mathrm{Fasting}\ \mathrm{Insulin}\right)}{22.5} $$

$$ \mathrm{HOMA}-\%\upbeta =\frac{20\times \mathrm{Fasting}\kern0.4em \mathrm{Insulin}}{\mathrm{Fasting}\kern0.4em \mathrm{Glucose}-3.5} $$

$$ \mathrm{The}\kern0.4em \mathrm{Matsuda}\kern0.4em \mathrm{Index}=\frac{10,000}{\sqrt{\left(\mathrm{Fasting}\kern0.4em \mathrm{glucose}\times \mathrm{Fasting}\kern0.4em \mathrm{Insulin}\right)\times \left(\mathrm{Mean}\kern0.4em \mathrm{glucose}\times \mathrm{Mean}\kern0.4em \mathrm{Insulin}\right)}} $$

Additional calculations for β-cell function include the insulinogenic index and oral disposition index (DI). Insulinogenic index was calculated as the change in insulin divided by change in glucose from 0 to 30 min. Oral disposition index was calculated as the product of (1/fasting insulin) x Insulinogenic index. The area under curve (AUC) for plasma glucose and serum insulin was calculated with the use of the trapezoidal rule method 0-120 min (OGTT measurements) for each time point (baseline, 3 months and 6 months). Data were analyzed with repeated measures of ANOVA (no adjustment or interaction was used for this model).

## Results

The participant flow diagram is presented in Fig. 1. A total of 884 were eligible for recruitment based on their HbA1c levels (between 5.6-6.4%) that indicate pre-diabetic phase and FINDRISC score (15-30 points) that indicates high to very high risk for T2DM. These participants were invited to the recruitment clinic at HMC, of whom 243 attended the visit. Following further screening of inclusion criteria, 209 participants were randomized to receive either vitamin D (*n* = 110) or placebo (*n* = 99) treatment. One hundred thirty-two participants successfully completed the trial, of which 57 constituted the vitamin D group and 75 the placebo group.

The baseline participant demographic characteristics were similar in both groups as shown in Table 1 apart from ethnicity. The vitamin D group included 40.4% participants of Arab ethnicity, whereas 64% were included in the placebo group. Participants were mainly male, 84 and 82.5%, with mean age 44.89 years (SD 8.88) and 45.51 years (SD 8.96) in the placebo group and vitamin D group, respectively. All participants were vitamin D deficient (< 30 ng/ml) with 87% having 25(OH) D serum levels below 20 ng/ml in the vitamin D group.

**Table 1:** Baseline participant’s characteristics

	Placebo (*n* = 75)	Vitamin D (*n* = 57)	*P*-value between groups
Age (years), *mean ± SD*	44.89 ± 8.88	45.51 ± 8.96	0.69
Gender (male), *n (%)*	63 (84.0)	47 (82.5)	0.81
Ethnicity (Arab), *n (%)*	48 (64.0)	23 (40.4)	0.007
25(OH) D (< 20 ng/ml), *n (%)*	55 (73.3)	45 (83.3)	0.18
Family history of diabetes (yes), *n (%)*	52 (69.3)	44 (77.2)	0.31
Family history of IHD (yes), *n (%)*	15 (20.3)	13 (23.2)	0.68
Family history of HTN (yes), *n (%)*	43 (58.1)	31 (55.4)	0.75
Physically active for at least 30 min per day (yes), *n (%)*	29 (38.7)	21 (37.5)	0.89
High blood glucose history (yes), *n (%)*	9 (12.0)	6 (10.5)	0.79
FINDRISC score, *mean ± SD*	12.01 ± 4.00	11.96 ± 4.05	0.94

FINDRISC score: A score calculated based on a questionnaire assessing the risk for diabetes

*Abbreviations*: *IHD* Ischemic heart disease, *HTN* Hypertension, *FINDRISC* Finnish diabetes risk score

Clinical characteristics were similar in the two groups (Table 2). HbA1c was 5.9% (SD 0.22) for the placebo and 5.9% (SD 0.19) for the vitamin D group. Participants were in the obese range in both groups with mean BMI 32.0 kg/m^2^ (SD 5.9) for the placebo and 30.0 kg/m^2^ (SD 6.2) for the vitamin D group. Serum 25(OH) D levels were slightly reduced in the placebo (− 0.88 ng/ml [95% CI: − 2.2, 0.44]) but almost doubled in the vitamin D group (19.4 ng/ml [95% CI: 16.4, 22.5], *P* < 0.001).

**Table 2:** Baseline values and mean changes for primary and secondary characteristics following 6-month vitamin D intervention

	Placebo (*n* = 75^a^)			Vitamin D (*n* = 57^a^)			*P*-value between groups^¶^	Adjusted *P*-value between groups^#^
	Baseline^b^	Change^d^	*p*-value within group^‖^	Baseline^b^	Change^d^	*P*-value within group^‖^		
Weight (kg)	91.3 ± 18.9	− 0.57 (− 1.53, 0.39)	0.24	86.3 ± 19.6	0.16 (− 0.73, 1.06)	0.71	0.28	−
BMI (kg/m^2^)	32.0 ± 5.9	− 0.19 (− 0.51, 0.13)	0.24	30.0 ± 6.2	0.05 (− 0.25, 0.35)	0.75	0.30	−
Waist Circumference (cm)	103.8 ± 12.6	− 0.16 (− 1.08, 0.76)	0.73	101.5 ± 11.0	− 0.39 (− 1.25, 0.47)	0.37	0.73	−
HbA1c (%)	5.9 ± 0.22	0.007 (− 0.10, 0.11)	0.90	5.9 ± 0.19	− 0.04 (− 0.12, 0.05)	0.37	0.54	−
HbA1c (mmol/mol)	41.3 ± 2.37	0.075 (− 1.07,1.22)	0.90	40.9 ± 2.02	− 0.40 (− 1.31,0.50)	0.37	0.54	−
SBP (mmHg)	129.3 ± 15.5	− 5.04 (− 8.27, − 1.81)	0.003	127.4 ± 12.8	−6.59 (− 10.44, − 2.74)	0.001	0.54	−
DBP (mmHg)	76.6 ± 12.0	− 0.84 (− 3.08, 1.40)	0.46	75.9 ± 9.9	− 4.87 (− 8.10, − 1.64)	0.004	0.036	−
RBC (× 10^6^ uL)	5.2 ± 0.51	− 0.08 (− 0.15, − 0.007)	0.03	5.2 ± 0.58	− 0.09 (− 0.19, 0.008)	0.07	0.84	−
Hemoglobin (g/dL)	14.5 ± 1.4	− 0.17 (− 0.34, − 0.004)	0.045	14.4 ± 1.4	0.16 (− 0.89, 1.22)	0.76	0.49	−
Hematocrit (%)	44.0 (42.0, 45.7)^c^	0.0 (− 1.4, 1.6)	0.46	43.2 (41.4, 45.8)	−1.0 (− 2.4, 1.4)	0.15	0.07	−
Total Cholesterol (mmol/L)	5.1 ± 0.95	− 0.08 (− 0.25, 0.09)	0.37	5.3 ± 0.86	− 0.32 (− 0.58, − 0.06)	0.02	0.11	−
HDL (mmol/L)	1.1 ± 0.23	− 0.04 (− 0.07, − 0.01)	0.005	1.1 ± 0.26	0.03 (− 0.08, 0.14)	0.58	0.15	−
LDL (mmol/L)	3.4 ± 0.86	− 0.08 (− 0.29, 0.12)	0.42	3.4 ± 0.80	− 0.15 (− 0.37, 0.06)	0.16	0.65	−
Triglyceride (mmol/L)	1.5 ± 0.74	0.16 (− 0.10, 0.43)	0.23	1.6 ± 0.84	0.16 (− 0.31, 0.63)	0.49	0.99	−
25 (OH) D (ng/ml)	17.0 ± 4.6	− 0.88 (− 2.20, 0.44)	0.19	14.9 ± 4.3	19.4 (16.4, 22.5)	< 0.001	< 0.001	−
PTH (pg/ml)	57.4 ± 28.3	7.7 (− 0.56, 15.9)	0.07	58.2 ± 19.4	− 3.1 (− 9.4, 3.1)	0.32	0.045	−
Serum Calcium (mmol/L)	2.25 ± 1.1	0.51 (− 0.16, 1.2)	0.13	1.99 ± 1.3	0.70 (0.06, 1.3)	0.03	0.67	−
Creatinine (Umol/L)	74.2 ± 11.3	− 4.1 (− 7.1, − 1.1)	0.008	73.0 ± 17.1	0.53 (− 4.6, 5.7)	0.84	0.11	−
CPK (U/L)	118.5 (83.5, 201.0)^c^	− 4.5 (− 32.8, 14.2)	0.36	124.5 (83.7, 185.7)	−2.0 (− 18.0, 15.0)	0.55	0.79	−
C-peptide (U/L)	2.1 (1.7, 3.1)^c^	0.18 (− 0.13, 0.59)	0.007	2.4 (1.9, 3.1)	0.15 (−0.29, 0.71)	0.12	0.84	−
SGOT (U/L)	21.0 (18.3, 26.8)^c^	−2.0 (− 7.0, 1.0)	0.011	22.0 (17.8, 27.3)	−3.0 (− 5.0, 2.0)	0.062	0.88	−
SGPT (U/L)	26.0 (19.0, 36.8)^c^	−3.0 (−9.0, 1.0)	< 0.001	29.0 (19.0, 42.0)	−3.0 (− 12.0, 3.0)	0.018	0.88	−
ALP (U/L)	73.7 ± 19.6	3.4 (−0.24, 7.0)	0.07	73.8 ± 17.7	−6.1 (− 10.1, −2.0)	0.004	0.001	−
Bilirubin (umol/L)	9.05 (7.2, 14.0)^c^	−0.75 (−2.4, 2.0)	0.699	11.3 (9.7, 15.0)	−1.85 (−5.2, 1.2)	0.006	0.039	−
Mean FPG (mmol/L)	6.3 ± 0.73	0.13 (−0.08, 0.35)	0.22	6.1 ± 0.63	0.30 (−0.09, 0.68)	0.13	0.41	0.434
2 h PCG (mmol/L)	9.3 ± 2.9	1.09 (0.44, 1.7)	0.001	9.2 ± 2.4	1.15 (0.44, 1.9)	0.002	0.90	0.768
Mean FPI (μU/ml)	9.8 ± 6.1	0.64 (−1.4, 2.7)	0.54	9.8 ± 5.0	− 1.4 (−2.6, −0.19)	0.024	0.11	0.137
2 h PCI (μU/mL)	64.9 (38.5, 138.3)^c^	1.2 (−30.0, 19.2)	0.717	98.6 (70.1, 180.3)	−23.3 (−70.9, 14.2)	0.06	0.22	0.882
Matsuda Index	2.9 (1.7, 4.1)^c^	0.21 (−0.84, 0.95)	0.731	2.2 (1.7, 3.4)	0.08 (−0.54, 0.99)	0.455	0.82	0.750
HOMA-IR	2.5 (1.7, 3.7)^c^	0.06 (−0.77, 0.86)	0.746	2.4 (1.5, 3.4)	−0.05 (− 0.94, 0.44)	0.455	0.45	0.326
HOMA- β	65.9 ± 44.3	−0.38 (−11.7, 11.0)	0.947	71.9 ± 36.8	−18.6 (−30.4, −6.8)	0.003	0.027	0.011
Disposition index	0.069 (0.034, 0.094)	0.0017 (−0.05, 0.013)	0.534	0.072 (0.05, 0.14)	−0.0087 (− 0.041, 0.022)	0.140	0.93	0.997
Insulinogenic index	0.58 (0.33, 0.98)	−0.054 (− 0.40, 0.15)	0.25	0.63 (0.45, 1.06)	−0.17 (− 0.30, 0.17)	0.018	0.79	0.586

*Abbreviations*: *BMI* Body mass index, *FPI* Fasting plasma insulin, *SBP* Systolic blood pressure, *DBP* Diastolic blood pressure, *RBC* Red blood cells, *PTH* Parathyroid hormone, *CPK* Creatine phosphokinase, *AST* Aspartate aminotransferase, *ALT* Alanine aminotransferase, *ALP* Alkaline phosphatase, *FPG* Fasting plasma glucose, *FPI* Fasting plasma insulin, *2 h PCG* 2 h post-challenge glucose, *2 h PCI* 2 h post-challenge insulin, *HOMA-IR* Homeostatic model of assessment - insulin resistance

‖ Data are analyzed by paired sample t-test or Wilcoxon signed rank test where appropriate (level of significance *P* ≤ 0.05)

^¶^ Data are analyzed by independent sample t-test (level of significance *P* ≤ 0.05)

^#^ Data are analyzed by ANCOVA using a general linear model and change values for each respective outcome with age, baseline BMI, gender and ethnicity as covariates

^a^ Initial sample size; the sample size was reduced for some variables due to missing values

^b^ Data are presented as mean ± SD

^c^ Data represented by median and inter-quartile range, Mann-Whitney U-test was used to compare median of the differences

^d^ Mean change; 95% CI or IQR where appropriate in parentheses

Measures of insulin sensitivity, with respect to the primary outcomes, did not differ between the placebo and vitamin D groups following the intervention, as presented in Table 2. Within both groups, the 2hPCG was significantly increased (1.15 mmol/L [95% CI: 0.44, 1.9], *P* = 0.002 for vitamin D and 1.09 mmol/L [95% CI: 0.44, 1.7], *P* = 0.001 for placebo). With regard to the secondary outcomes, also presented in Table 2, fasting plasma insulin (mean FPI) was decreased in the vitamin D group (− 1.4 μU/ml [95% CI: − 2.6, − 0.19], *P* = 0.024) but measures did not differ in group comparison (*P* = 0.11). For the β-cell function, a significant change was observed for HOMA-β between the two groups (− 18.6 [95% CI: − 30.4, − 6.8] vs − 0.38 [95% CI: − 11.7, 11.0], *P* = 0.027) and significance persisted after adjusting for age, baseline BMI and gender (*P* = 0.010). HOMA-β significantly decreased within the vitamin D group (*P* = 0.003). Similarly, a slight decrease in insulinogenic index was observed for the same group (*P* = 0.018). An expected but not significant reduction of parathyroid hormone (PTH) was found in the vitamin D group (Table 2). In the same group, serum calcium slightly increased (0.70 mmol/L [95% CI: 0.06, 1.3], *P* = 0.03) and total cholesterol decreased (− 0.32 μg/ml [95% CI: − 0.58, − 0.06], *P* = 0.02) but both measures did not differ between the two groups. Both the systolic (*P* < 0.001) and diastolic pressure (*P* = 0.004) decreased within the treatment group but only diastolic pressure was significant in group comparison (*P* = 0.036). Statistical differences between the vitamin D and placebo group were also observed for alkaline phosphatase (ALP) (− 6.1 U/L [95% CI: − 10.1, − 2.0] vs 3.4 [95% CI: − 0.24, 7.0], *P* = 0.001) and bilirubin (− 1.85umol/L [IQR -5.2, 1.2] vs − 0.75 [IQR -2.4, 2.0], *P* = 0.039).

**Fig. 2:**
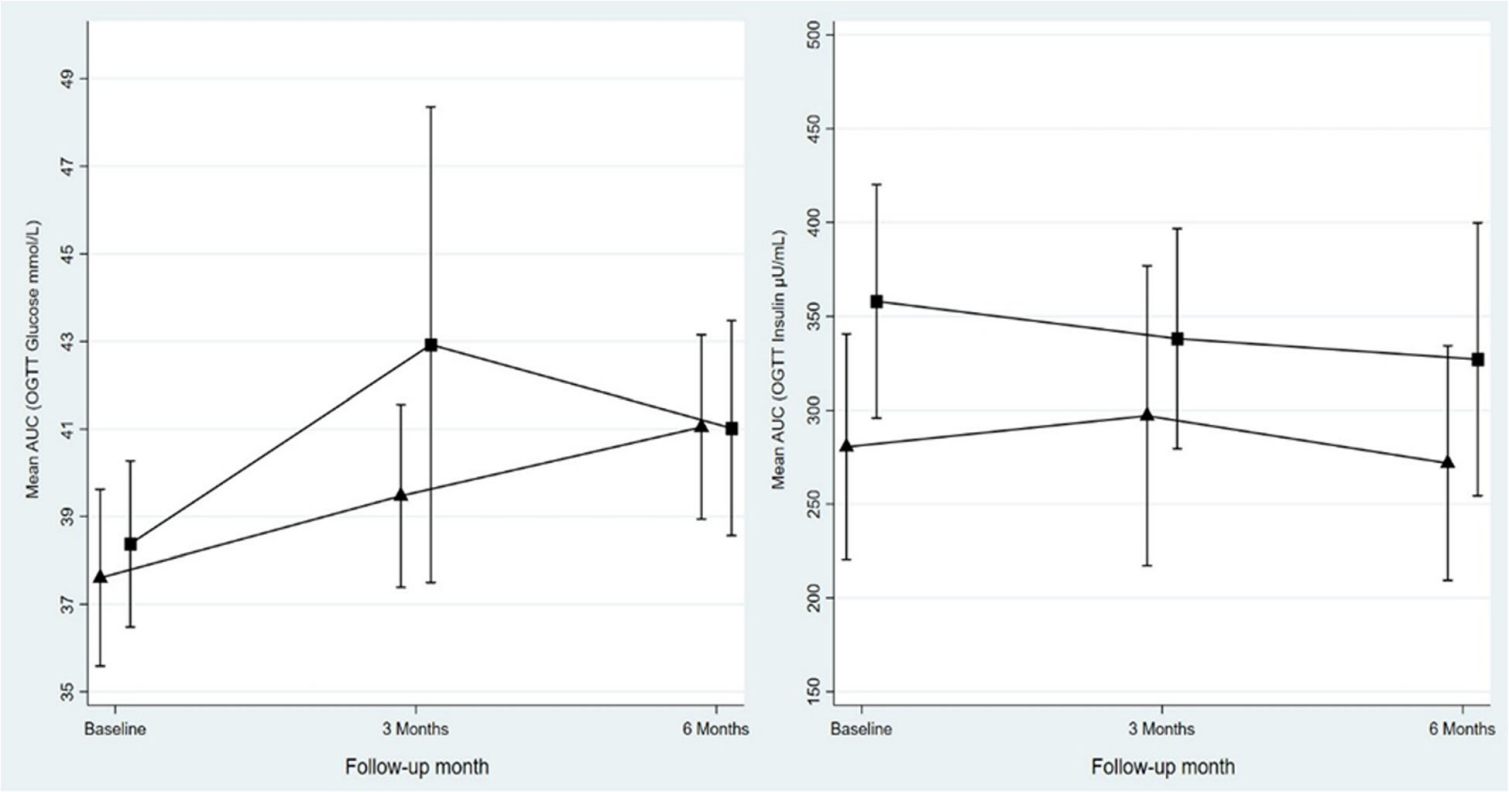
Effect of vitamin D supplementation on mean area under curve (AUC) for glucose (mmol/L) and insulin (μU/mL) from baseline to end point for placebo (black triangles) and vitamin D (black squares) groups

As shown in Fig. 2, the AUC for glucose significantly increased within both groups (*P* = 0.0146, *P* < 0.001) from baseline to endpoint (6 months). The opposite was observed for the AUC for insulin (*P* < 0.001) from baseline to endpoint. Significance persisted for both aforementioned measures in group comparison (Additional file 1: Table S1).

In additional bivariate association analysis no significant correlations were found between vitamin D change and clinical characteristics from baseline to 6 months intervention (Additional file 2: Table S2). Multivariable linear regression analyses indicated no significant associations between primary and secondary outcomes and vitamin D change from baseline to endpoint (data not shown).

## Discussion

Findings from our study showed that vitamin D supplementation over a 6-month period with 30,000 IU vitamin D per week markedly increased 25(OH) D levels in the intervention group. Supplementation had no effect on glucose tolerance or insulin sensitivity in our pre-diabetic and severely vitamin-deficient population. Although we observed an impaired β-cell function suggested by the significant reduction in HOMA-β within the vitamin D group and in group comparison, further analysis did not support this observation. The Insulinogenic and Disposition Indices were not significantly different between the two groups. In addition AUC for glucose and insulin was found to be significant within both groups as well as group comparison. Thus, It is unclear whether the changes in β-cell function can solely be attributed to vitamin D supplementation.

Although heterogeneity across the studies, particularly in the study population characteristics (e.g. sample size, vitamin D status, stage of diabetes) may confound comparison, our findings seem to be in accordance with most available intervention studies. Moreira-Lucas et al. recently found no effect of vitamin D supplementation on diabetes risk outcome measures, when participants received 28,000 IU of vitamin D in fortified cheese weekly [19]. Similarly, Davidson et al. observed that supplementation with high dose of vitamin D weekly (88,865 IU) for a year did not affect glucose metabolism or insulin sensitivity but HbA1c levels were reduced in the vitamin D group [18]. Although participants were pre-diabetics they were not vitamin D-deficient, which is an essential factor to evaluate the efficacy of supplementation on glycemic measurements [27]. In another trial by Wagner et al. with short-term intervention (8 weeks) and small sample size (*n* = 44), supplementation did not affect glucose tolerance or insulin sensitivity [20]. A hyperglycemic clamp was used to calculate the disposition index (DI) in one of the trials [20] while insulin secretion was assessed by OGTT-based indices in both aforementioned studies.

Consistent to our findings were also two recent meta-analyses of RCTs [28, 29]. The first found no effect of vitamin D supplementation (oral or by injection) on insulin sensitivity, measured by HOMA-IR, in participants with normal glucose tolerance, pre-diabetes or type 2 diabetes [28]. Results remained unchanged when analysis was restricted to pre-diabetic participants. In the second review, where participants were only pre-diabetics, vitamin D supplementation did not alter insulin resistance but in a subgroup analysis with baseline vitamin D < 50 nmol/L, FPI and HbA1C were significantly reduced following supplementation [29].

In contrast, some studies have found a favorable effect of vitamin D intervention on glycaemic measures. In a study by von Hurst et al. 4000 IU of vitamin D daily for 6 months reduced insulin resistance and fasting insulin, in an insulin-resistant and vitamin D deficient population. Although these findings were promising, the study included only women and HOMA-IR was used as an insulin sensitivity measure, which reflects liver and not whole body insulin sensitivity [11]. In another intervention study conducted by Mitri et al., low dose, short-term vitamin D supplementation (2000 IU daily for 16 weeks) improved β-cell function and had a marginal effect on attenuating the rise in HbA1c [30]. However, this study only adjusted for seasonal changes and lacked other important confounding factors such as age, BMI and gender. Nagpal et al. also reported an improvement in postprandial insulin sensitivity although the clinical trial included only men who were obese and non-diabetic [31]. In addition, Nikooyeh et al., demonstrated a significant reduction in fasting glucose, HbA1c and insulin resistance (HOMA-IR) when participants with Type 2 Diabetes received vitamin-D fortified yoghurt [32].

In our study, although we were able to include severely vitamin D-deficient and pre-diabetic participants in a relatively large study sample, we still could not observe any correlations between the outcomes and the increase in 25(OH) D level in the intervention group. It is worth mentioning that, although there was a substantial increase in 25(OH) D concentrations, study participants only achieved serum 25(OH) D levels of approximately 35 ng/mL after 6 months intervention period. This is just above the lowest level of the normal range of vitamin D status and it could be attributed to the absorption of the supplements in participants with high BMI [33]. Most participants in the vitamin D group were in the obese or overweight range (BMI ≥ 30 Kg/m^2^) and this may be associated with smaller increases in 25(OH) D concentrations following the supplementation [33]. In this case some may argue that the dose of supplementation should exceed the 4000 IU/day, especially in extremely deficient participants. According to available evidence vitamin D supplementation dose between 4000 IU and 10,000 IU per day has shown no toxic effects [27, 34], however in the current study supplementation was kept to the lower adverse effect dose as more research is needed about the efficacy and safety of higher doses.

When comparing the vitamin D and placebo groups following the intervention period, a decrease was found in PTH in the vitamin D group. This is an expected observation since a reduction of parathyroid hormone has been associated with vitamin D supplementation [35]. Similarly, bilirubin levels were reduced in both groups with more pronounced reduction in the vitamin D group. The net effect of vitamin D supplementation is likely to be obstructed by the decreased levels of bilirubin. Animal studies have shown that bilirubin treatment in mice reduced blood glucose levels and increased insulin sensitivity [36]. Recent meta-analysis in human studies also confirm the potential protective role of bilirubin against the risk of diabetes [37].

Strengths of our study include the relatively large sample size and population characteristics. Participants were at pre-diabetic stage and most of them were severely vitamin D deficient. In addition, we were able to assess β-cell function by multiple measurements which has only been examined by limited number of interventional studies. The outcomes of these measurements could be attributed to our exclusively pre-diabetic population which excludes any bias introduced by mixed diabetes status study sample. In group comparison between baseline and end-point we were able to adjust for multiple confounding factors including age, baseline BMI and gender but did not adjust for seasonal changes. However, we would not expect an important effect from the seasonal change since Qatar has vast sunlight throughout the year. The study population included participants of Arab or Asian ethnicity and thus the present findings are population specific and cannot be easily extrapolated to the general population. Some may also argue that 6 months of vitamin D supplementation was not enough to observe the effect on glycemic parameters especially for participants like ours with high BMI. Compliance regarding the vitamin D intake may have introduced bias since it was self-administered. Finally, 77.2% of the participants in the vitamin D group had family history of diabetes, which could have contributed genetically in attenuating any effects of vitamin D on glycemic measures as it is well known that diabetes is highly inheritable [38].

## Conclusions

According to our findings, supplementation with vitamin D did not improve insulin sensitivity, insulin secretion, or glucose tolerance in our pre-diabetic and highly vitamin D deficient population. Larger and multicenter intervention studies, in such populations, with longer duration are required to examine the role of vitamin D, combined with other lifestyle interventions targeting risk factors and surrogate markers of T2DM.

## Supplementary information

**Additional file 1: Table S1.** Effect of vitamin D supplementation reflected in OGTT measures overtime for the two groups.

**Additional file 2: Table S2.** Bivariate associations between vitamin D change and clinical characteristics from baseline to 6 months intervention in the vitamin D group.

## Data Availability

The datasets used and/or analysed during the current study are available from the corresponding author on reasonable request.

## Abbreviations

[25-(OH)D]: Vitamin D
2 h PCG: 2 h Post-challenge glucose
2 h PCI: 2 h Post-challenge insulin
ALP: Alkaline phosphatase
ALT: Alanine aminotransferase
AST: Aspartate aminotransferase
AUC: Area under the curve
BMI: Body mass index
CLIA: Chemiluminescence immunoassay
CMIA: Chemiluminescent Micro Particle Immunoassay
CPK: Creatine phosphokinase
DBP: Diastolic blood pressure
FINDRISC: Finnish Diabetes Risk Score
FPG: Fasting plasma glucose
FPI: Fasting plasma insulin
HMC: Hamad Medical Corporation
HOMA- β: Homeostatic Model of Assessment- Beta
HOMA-IR: Homeostatic Model of Assessment - Insulin Resistance
HTN: Hypertension
IHD: Ischemic heart disease
OGTT: Oral glucose tolerance test
PTH: Parathyroid hormone
RBC: Red blood cells
RCTs: Randomized control trials
SBP: Systolic blood pressure
T2DM: Type 2 diabetes
ULN: Upper limit of normal
