# Supplementary material for: The effect of vitamin D supplementation on the glycemic control of pre-diabetic Qatari patients in a randomized control trial
Source: BMC Nutr. 2019 Oct 10;5:46. doi: 10.1186/s40795-019-0311-x (PMC7050821; doi:10.1186/s40795-019-0311-x)
Supplement: Supplementary file 1 — Additional file 1: Table S1. Effect of vitamin D supplementation reflected in OGTT measures overtime for the two groups. [file 40795_2019_311_MOESM1_ESM.docx]

Additional file 1: Table S1. Effect of vitamin D supplementation reflected in OGTT measures overtime for the two groups.

|  | Placebo | | | | Vitamin D | | | | |
| --- | --- | --- | --- | --- | --- | --- | --- | --- | --- |
|  | Baseline^*^ | 3 months^*^ | 6 months^*^ | *P*-value  within group^†^ | Baseline^*^ | 3 months^*^ | 6 months^*^ | *P*-value within group^†^ | *P*-value between groups^†^ |
|  | n=69 | n=69 | n=69 |  | n=48 | n=48 | n=48 |  |  |
| Mean AUC glucose | 40.8 ± 8.8 | 42.7 ± 9.1 | 44.3 ± 9.3 | < 0.0001 | 41.3 ± 7 | 46.1 ± 19.6 | 44.2 ± 9.1 | 0.0123 | < 0.0001 |
|  | n=36 | n=36 | n=36 |  | n=31 | n=31 | n=31 |  |  |
| Mean AUC insulin | 285.7 ± 186.4 | 303.1 ± 247.7 | 276.8 ± 193.1 | < 0.0001 | 363 ± 178.3 | 342.6 ± 167.5 | 331.4 ± 207.8 | < 0.0001 | < 0.0001 |
| Abbreviations: AUC, Area Under Curve.  ^*^ Data are presented as mean ± SD.  ^†^ Data analyzed using repeated measures of ANOVA. | | | | | | | | | |
