# Supplementary material for: The effect of vitamin D supplementation on the glycemic control of pre-diabetic Qatari patients in a randomized control trial
Source: BMC Nutr. 2019 Oct 10;5:46. doi: 10.1186/s40795-019-0311-x (PMC7050821; doi:10.1186/s40795-019-0311-x)
Supplement: Supplementary file 2 — Additional file 2: Table S2. Bivariate associations between vitamin D change and clinical characteristics from baseline to 6 months intervention in the vitamin D group. [file 40795_2019_311_MOESM2_ESM.docx]

Additional file 2: Table S2. Bivariate associations between vitamin D change and clinical characteristics from baseline to 6 months intervention in the vitamin D group.

|  | Vitamin D group | | |
| --- | --- | --- | --- |
|  | N | r^*^ | *p*-value ^†^ |
| Age (years) | 53 | 0.144 | 0.304 |
| Gender (male/female) | 53 | 0.112 | 0.425 |
| Weight (kg) | 53 | 0.217 | 0.118 |
| BMI (kg/m^2^) | 53 | 0.222 | 0.110 |
| Waist Circumference (cm) | 51 | 0.272 | 0.053 |
| HbA1c (%) | 53 | -0.081 | 0.564 |
| FPI (uU/ml) | 36 | 0.069 | 0.691 |
| SBP (mmHg) | 53 | -0.134 | 0.339 |
| DBP (mmHg) | 53 | -0.067 | 0.632 |
| RBC (x10^6^ uL) | 52 | -0.215 | 0.126 |
| Hemoglobin (g/dL) | 53 | 0.021 | 0.882 |
| Hematocrit (%) | 53 | 0.009 | 0.952 |
| Total Cholesterol (mmol/L) | 53 | 0.167 | 0.233 |
| HDL (mmol/L) | 53 | -0.082 | 0.560 |
| LDL (mmol/L) | 50 | 0.098 | 0.497 |
| Triglyceride (mmol/L) | 53 | 0.124 | 0.376 |
| PTH (pg/ml) | 47 | -0.198 | 0.181 |
| Serum Calcium (mmol/L) | 37 | -0.126 | 0.457 |
| Creatinine (Umol/L) | 53 | 0.129 | 0.358 |
| WBC (x10^3^ uL) | 53 | 0.110 | 0.432 |
| Platelets (x10^3^ uL) | 52 | -0.057 | 0.688 |
| CPK (U/L) | 48 | -0.075 | 0.611 |
| C-peptide (U/L) | 47 | -0.090 | 0.547 |
| GOT (U/L) | 32 | 0.235 | 0.196 |
| GPT (U/L) | 52 | 0.0001 | 0.997 |
| ALP (U/L) | 53 | -0.082 | 0.558 |
| Bilirubin (umol/L) | 53 | -0.145 | 0.302 |
| Mean FPG (mmol/L) | 53 | 0.072 | 0.607 |
| 2h PCG (mmol/L) | 50 | -0.055 | 0.705 |
| Mean FPI (uU/ml) | 36 | 0.069 | 0.691 |
| 2h PCI (uU/mL) | 34 | -0.022 | 0.902 |
| Matsuda Index | 36 | 0.117 | 0.303 |
| HOMA-IR | 36 | 0.007 | 0.969 |
| HOMA- β | 34 | -0.193 | 0.273 |
| ^*^ Spearman’s rho indicating strength of association.  ^†^ Data are analyzed by Spearman’s rank correlation; level of significance *p*≤0.05. | | | |
